# Antibiotic Resistance and Therapeutic Efficacy of *Helicobacter pylori* Infection in Pediatric Patients—A Tertiary Center Experience

**DOI:** 10.3390/antibiotics12010146

**Published:** 2023-01-11

**Authors:** Felicia Galoș, Cătălin Boboc, Mara-Ioana Ieșanu, Mălina Anghel, Andreea Ioan, Elena Iana, Maria Teodora Coșoreanu, Anca Andreea Boboc

**Affiliations:** 1 Department of Pediatrics, Carol Davila University of Medicine and Pharmacy, 020021 Bucharest, Romania; 2 Department of Pediatrics, Marie Curie Emergency Children’s Hospital, 041451 Bucharest, Romania; 3 Department of Functional Sciences, Carol Davila University of Medicine and Pharmacy, 020021 Bucharest, Romania

**Keywords:** *Helicobacter pylori*, cultures, antibiotic resistance, children, sequential therapy

## Abstract

One of the most widespread bacterial infections worldwide, *Helicobacter pylori* is thought to affect almost half of the world’s population. Due to rising antibiotic resistance, treatment should be tailored according to antibiotic susceptibility testing (AST). This study aims to evaluate *Helicobacter pylori* antibiotic resistance and its therapeutic efficacy in children. We conducted a prospective, single-center study, that evaluated 68 children referred for upper gastrointestinal endoscopy (UGE) following chronic dyspeptic syndrome. Each patient underwent multiple biopsies to perform bacterial cultures with AST and histopathological examinations for the diagnosis. Patients without antibiotic resistance or negative cultures received a 10-day sequential therapy, while the others had the antibiotic regimen tailored based on AST. Fifty-nine patients with a positive biopsy-based diagnosis (24 males) were finally included. Bacterial cultures with AST were positive for 13 patients (22.03%) and the antibiotic resistance for clarithromycin was 15.38%. Fifty-seven patients were administered sequential therapy with an eradication rate of 94.73%. Clarithromycin-resistant patients were successfully treated with 10-day triple therapy of esomeprazole, amoxicillin, and metronidazole. Although bacterial cultures had a low positivity rate, sequential therapy had a successful eradication rate. Further studies are necessary to better assess *Helicobacter pylori* antibiotic resistance to provide tailored treatment and identify children that need closer monitoring.

## 1. Introduction

In 1982, Warren and Marshall isolated for the first time *Helicobacter pylori (H. pylori)* from gastric biopsies of patients with chronic gastritis and peptic ulcers [1]. As a category one carcinogen since 1994, *H. pylori* is currently regarded as the most prevalent etiologic agent of infection-related malignancies, which represent 5.5% of the global cancer burden [2,3]. The association of gastric cancer, one of the most frequent causes of death worldwide, with a treatable etiological factor, led to a profound impact on cancer research. Moreover, the World Health Organization nominated *H. pylori* as a pathogen of high priority for novel antimicrobial drug findings due to its high rate of antibiotic resistance, especially to clarithromycin [4].

*H. pylori* infection affects more than 50% of people worldwide, and cross-sectional studies suggest that infection almost invariably occurs within the first five years of life [5]. Less than 10% of children in developed countries are infected by the age of ten, reflecting a substantial decline in infection over the past 30 years. However, prevalence rates of up to 80% in children under the age of five have been reported in underdeveloped countries [6]. This high prevalence is generally related to poorer socioeconomic status and level of hygiene [7]. In Romania, a developing country, the prevalence of *H. pylori* infection is high, around 68% in adults [7,8] and ranging from 33% to 45% in children [9,10,11].

*H. pylori* are responsible for the onset of several pediatric gastric pathologies ranging from mild gastritis to malignancies. *H. pylori* can trigger gastritis and peptic ulceration, while long-term infections can lead to gastric adenocarcinoma and mucosa-associated lymphoid tissue (MALT) lymphoma [12]. Moreover, implications in other gastrointestinal pathologies (e.g., gastroesophageal reflux) or extra-digestive disorders (e.g., iron-deficiency anemia, chronic idiopathic thrombocytopenia, and growth failure) were recently discovered [12,13]. Children may experience non-specific symptoms of peptic ulcer disease caused by *H. pylori*, such as epigastric pain, especially after meals, night-time waking, unexplained nausea or vomiting, anorexia, and hematemesis [14].

When considering the diagnostic strategies for *H. pylori* infection in children, the Joint European Society for Pediatric Gastroenterology, Hepatology, and Nutrition (ESPGHAN) and North American Society for Pediatric Gastroenterology, Hepatology, and Nutrition (NASPGHAN) guidelines support upper gastrointestinal endoscopy (UGE) with biopsies as the most reliable method [15]. From the biopsy samples, two diagnostic techniques are recommended, bacterial cultures with antibiotic susceptibility testing (AST) and histopathological examination with an additional biopsy-based test (e.g., rapid urease test—RUT, polymerase chain reaction, or fluorescent in situ hybridization) [15]. For the assessment of histopathological changes, the guidelines recommend the use of the Updated Sydney Classification for gastritis [16,17]. 

In children, there are only a few antibiotics effective for treating *H. pylori* infection [18]. The treatment is usually empiric and includes antibiotic regimens comprising amoxicillin, clarithromycin, or metronidazole [15]. Levofloxacin and tetracycline are used mainly in adults for *H. pylori* eradication. Among pediatric patients, levofloxacin can be given only to adolescents due to possible negative effects on cartilage and teeth, while tetracycline is recommended for children older than 8 years old [15]. Besides the scarce drug availability in children, the rising antibiotic resistance enhances the difficulty in treating pediatric patients with *H. pylori.* Antibiotic resistance varies widely by area; for instance, it is more prevalent in Central/Western and Southern European countries than in Northern European nations [19]. Additionally, studies from early 2020 report an incidence of 23.5% [20], while more recent ones describe resistance in more than two-thirds of the patients infected with *H. pylori* [21]. As a result, physicians are facing a significant challenge in treating *H. pylori* infection in children. Thus, it is important to profile local or population-specific antibiotic resistance to determine the best course of treatment for children with *H. pylori*.

The current study aims to first, isolate *H. pylori* in cultures from pediatric gastric biopsy samples and test their susceptibility to amoxicillin, clarithromycin, metronidazole, and levofloxacin. Secondly, we aim to assess the efficacy of the sequential therapy in the pediatric population from our tertiary center during a specific timeframe. 

## 2. Material and Methods

### 2.1. Patients Selection

We performed a prospective, observational, single-center study that included 68 pediatric patients that underwent UGE at Marie Curie Emergency Children’s Hospital in Bucharest (Romania) between July 2015 and March 2018. These patients were referred by their physicians for UGE due to chronic dyspeptic syndrome. All the patients were screened at the beginning of the study for *H. pylori* and all of them presented a positive stool antigen test. The used test was the OnSite *H. pylori* Ag Rapid Test, which was validated using internal and external controls. 

Depending on their age and level of understanding, pediatric patients or parents filled out a questionnaire to obtain information about each patient’s demographics and personal and family history. Patients’ demographic data included their age, gender, and place of residence (urban or country area). History of *H. pylori* infection, as well as previous therapies, were noted. Prior infectious diseases among the other members of the family were also assessed. Patients were questioned regarding the onset and duration of the GI symptoms and the use of different drugs (e.g., proton pump inhibitors—PPIs, H_2_ receptor antagonists, non-steroidal anti-inflammatory drugs, or corticosteroids). Smoking status and alcohol consumption among adolescents were evaluated. 

Exclusion criteria consisted of the use of PPIs, H_2_ receptor antagonists, antibiotics, non-steroidal anti-inflammatory drugs, and/or corticosteroids two weeks prior to the beginning of the study. This recommendation was similar to other studies [19]. By altering the pH, PPIs can indirectly interfere with *H. pylori* distribution in the stomach. The antrum is the most affected part of the stomach as PPIs can almost eradicate *H. pylori* from this area [22]. Thus, to elude false negative results, it is recommended to avoid these drugs at least two weeks before the endoscopy [19,23]. Additionally, a history of GI surgery (except polypectomy and appendectomy), concomitant severe disease (heart, lungs, kidneys, and endocrine diseases), and smoking or alcohol consumption were also considered exclusion criteria. 

Sixty-eight patients met the aforementioned criteria and were included in the initial study. However, only 59 cases were considered eligible for having a positive diagnosis. The *H. pylori* diagnosis was based on invasive gastric biopsy techniques, including positive bacterial cultures, or histopathological findings of *H. pylori* scored using the Updated Sydney System [15,17]. The present study accepted positive bacterial cultures and histologic *H. pylori* findings as the “gold standard” for the diagnosis. According to the recommendations, the detection of *H. pylori* in at least one of these two investigations is accepted as *H. pylori*-positive diagnosis [15]. Negative results in both cultures and histology excluded the *H. pylori* infection. Thus, for the final analysis, only 59 patients were considered. 

The protocol was accepted by the Marie Curie Emergency Children’s Hospital’s ethics committee (197/27 May 2015 and 627/26 May 2017), and the study was carried out in accordance with the Declaration of Helsinki. Prior to the UGE, the patients’ parents or legal guardians provided their informed consent.

### 2.2. Upper Gastrointestinal Endoscopy

All 68 patients underwent UGE, and biopsy specimens were taken for histopathologic examination (one sample from the antrum and one from the corpus) and bacterial cultures (two samples from the antrum). One additional sample from the antrum was used occasionally for the RUT. The samples were placed into distinct vials that had been previously marked and contained the appropriate medium for each test. The procedure was performed in patients with a minimum of 10 h of fasting, under general anesthesia or conscious sedation. During the entire procedure, vital signs were continuously monitored. No incidents or adverse effects were noted during or after the UGE. 

### 2.3. Helicobacter pylori Cultures and Antibiotic Susceptibility Testing

The biopsy samples obtained for bacterial culture were transported in a special *H. pylori* transport medium (i.e., Portagerm pylori from BioMérieux SA, Marcy l’Etoile, France), and inoculated after a few hours onto selective medium Pylori Agar (BioMérieux Italia). For 72 h, the plates were incubated at 37 °C in a microaerobic environment. After incubation, Gram stain and oxidase, catalase, and urease tests were used to identify the *H. pylori* colonies. To perform an E-test on pylori agar, suspensions from the primary plates were prepared in a sterile solution. An E-Test strip (E-Test; AB Bio disk, Solna, Sweden) was placed onto each separate plate and immediately incubated in a microaerobic atmosphere at 37 °C for another 72 h. This procedure involved streaking an agar plate in three directions with a swab dipped into each bacterial suspension to create a lawn of growth. Isolated strains were screened for several antibiotic resistance (e.g., amoxicillin, clarithromycin, metronidazole, and levofloxacin) according to the recommendations of the European Committee on Antimicrobial Susceptibility Testing. 

### 2.4. Histopathological Examination

Gastric corpus and antrum biopsy samples were preserved in a 10% formaldehyde solution for further analysis. Following that, the gastric mucosa processing steps of dehydration and paraffin embedding were carried out as usual. For the histologic examination, two stains were used, namely hematoxylin–eosin and Giemsa stains. 

Hematoxylin–eosin is an inexpensive, rapid, simple test that allows evaluation of *H. pylori*’s presence and its complications (e.g., intestinal metaplasia, neoplasms) [24]. However, this method presents some disadvantages, not being able to detect *H. pylori* in mild gastritis, when the bacterial amount is low, or when the patient received PPIs or antibiotics [25]. Thus, Giemsa is recommended as an ancillary stain in case the first one fails to identify the bacterium [24,25]. Giemsa stain is also a simple, rapid, and inexpensive test, that has superior sensitivity when compared to hematoxylin–eosin [24,25].

In the histology section, *H. pylori* were identified as short, spiraled, or curved bacilli lying on the mucus layer or epithelial surface [1]. The Updated Sydney System was used to characterize the morphological features of *H. pylori*-associated gastritis from the biopsies [16,17]. This classification evaluates the mucosal degree of inflammation, level of activity, and the presence of atrophy or intestinal metaplasia. According to this system, the density of *H. pylori* infection can be semi-quantitatively graded on a scale from 0 to 3 (i.e., none, mild, moderate, or severe) [17]. 

### 2.5. Therapeutic Regimens

Patients with a positive *H. pylori* diagnosis received adequate treatment for bacterial eradication. The antibiotic therapy was tailored based on AST where possible. In case of no antibiotic resistance or negative cultures, patients received a 10-day sequential therapy with a standard dosing regimen according to the recommendations [15]. Patients were administered PPI (i.e., esomeprazole) with amoxicillin for 5 days, followed by PPI with clarithromycin and metronidazole for another 5 days. The antibiotic dosage was adjusted according to body weight. Patients having less than 15 kg weight received the following recommended doses: esomeprazole 1 mg/kg/day, amoxicillin 50 mg/kg/day, clarithromycin 15 mg/kg/day, and metronidazole 20 mg/kg/day, divided equally every 12 h. For the other patients, the administered doses were as follows: esomeprazole—20 mg (15–24 kg), 30 mg (25–34 kg), 40 mg (>35 kg), amoxicillin—500 mg (15–24 kg), 750 mg (25–34 kg), 1000 mg (>35 kg), clarithromycin—250 mg (15–24 kg), 500 mg (>25 kg), and metronidazole—250 mg (15–24 kg), 500 mg (>25 kg), twice daily [15]. Patients resistant to clarithromycin were treated with 10-day targeted triple therapy of esomeprazole, amoxicillin, and metronidazole using the recommended doses. 

Following the guidelines [15], at four to six weeks after antibiotic treatment completion, the patients had their symptoms evaluated by questionnaire, were physically examined, and were tested for *H. pylori* infection using the stool antigen test.

### 2.6. Data Analysis

The statistical analysis was performed using IBM SPSS Statistics 26. Continuous data with normal distribution were expressed as the mean ± standard deviation (SD). The normality was tested using the Shapiro–Wilk test. Differences and relationships between variables were analyzed using Fisher’s Exact test for low expected frequencies. A *p*-value < 0.05 was considered statistically significant for all the analyzed parameters, with a 95% confidence level for intervals. Sensitivity and specificity for *H. pylori* cultures and histologic examination were also calculated. 

## 3. Results

**Figure 1:**
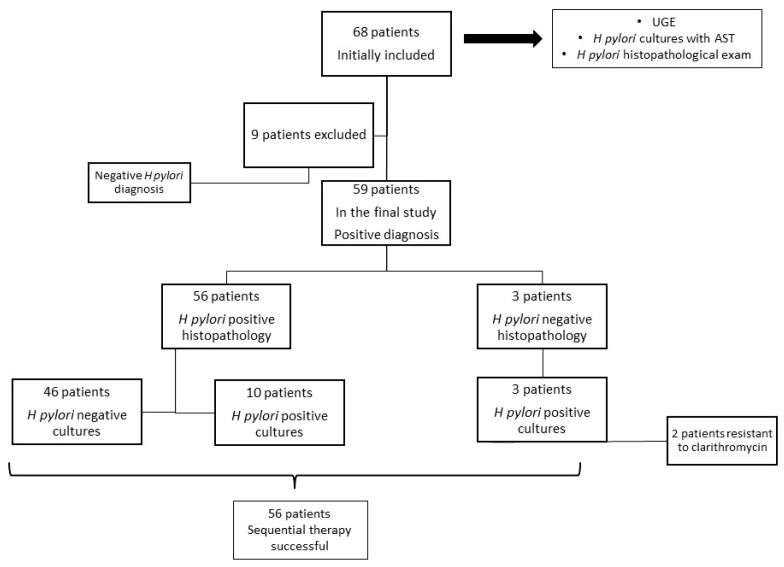
Flowchart of the selection of the patients included in the study. Abbreviations: UGE, upper gastrointestinal endoscopy; *H. pylori, Helicobacter pylori;* AST, antibiotic susceptibility testing.

All 68 patients enrolled in the study underwent UGE with biopsies. However, nine individuals were excluded due to negative results in both cultures and histology, indicating an 86.76% positivity rate (Figure 1). 

For the final analyses, only 59 patients (86.76%) were included based on their positive *H. pylori* diagnosis. Thirty-five patients were females (59.32%) and 24 males (40.67%) and the age ranged between 2 years and 5 months and 17 years and 8 months, with a mean value of 11 years and 7 months ± 4 years and 5 months. In Table 1, the demographic and clinical characteristics of the patients included in the study are highlighted. Ten patients (16.94%) had a family history of peptic ulcer disease, 26 children (44.06%) presented symptoms for more than six months, and 22 patients (37.28%) were previously treated for *H. pylori*. The mean age of the patients who were previously treated was 11.63 ± 4.45 years old and 11.91 ± 4.85 years old for those who have not received any therapies in the past (*p* = 0.77, 95% CI −1.84: 2.43). Additionally, there was a statistically significant association between the severity of mucosal damage during endoscopy and the existence of a previous therapy against the infection (*p* = 0.01). Seven of the eight patients (87.50%) with normal endoscopic mucosa were previously treated (Table 2).

The mean duration between the onset of symptoms and the effective diagnosis in patients having a family history of upper GI diseases was 6.31 ± 6.80 months, while for those without a family history tended it to be higher at 8.14 ± 7.27 months (*p* = 0.41, 95% CI −6.70: 3.02). 

**Figure 2:**
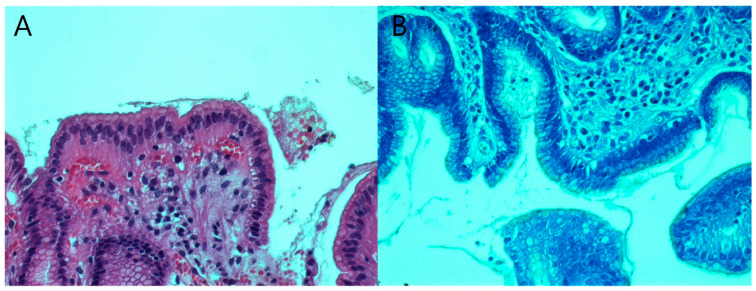
Microscopic view of *Helicobacter pylori* in antral gastric biopsy stained with (**A**) hematoxylin–eosin, and (**B**) Giemsa. (**A**) 200× histological section of hematoxylin–eosin-stained antral gastric mucosa with inflammatory lymphoplasmacytic infiltration and vascular congestion; *Helicobacter pylori* can be seen within the mucus layer at the surface of gastric mucosa; (**B**) 400× histological section of Giemsa-stained antral gastric mucosa with *Helicobacter pylori* colonization of the gastric glands, depicted as minuscule curved structures.

The bacterial cultures were positive and the corresponding antibiotic susceptibility tests were available only for 13 patients, representing 22.03% of the total number of patients. Two of the bacterial cultures presented clarithromycin resistance. Both patients were previously administered *H. pylori* infection treatment. Thus, the clarithromycin resistance in our study was 15.38%. In three of these cases, although the cultures were positive, the bacteria could not be identified through the histological exam. The specificity for *H. pylori* culture in our study was 93.87%, but the sensitivity was low, only 22.03%. Histological examination was able to identify the presence of *H. pylori* in 56 patients (94.91%) (Figure 2). The specificity, in this case, was 94.23% and the sensitivity was 94.91%. 

Additionally, we found a statistically significant correlation between the density of *H. pylori* in the histological exam and the number of positive bacterial cultures (*p* = 0.01), but under the reserve of a limited number of positive cultures. Moreover, we also checked the relationship between the activity of gastritis and the density of *H. pylori* in the histopathological exam and found a statistically significant correlation between them as we have previously showed in a smaller cohort. According to our results, this substantial difference between the groups allowed us to conclude that the degree of gastritis activity was related to the histologic *H. pylori* density (*p* = 0.03) (Table 3).

Regarding the treatment, 57 patients were administered sequential therapy. The two patients resistant to clarithromycin were treated with 10-day targeted triple therapy consisting of esomeprazole, amoxicillin, and metronidazole. The eradication rate for sequential therapy was 94.73% since three children presented with positive fecal *H. pylori* antigen after treatment. 

## 4. Discussion

Despite evidence that *H. pylori* prevalence is declining in developed countries, the infection is nonetheless ubiquitous with important morbidity and mortality rates [26]. *H. pylori* can commonly be acquired during childhood and persist throughout life with rare spontaneous remission [27]. One of the main concerns nowadays is related to *H. pylori* eradication failure and antibiotic resistance [28]. Moreover, antibiotic misuse is one of the primary causes of this trend. As mentioned, *H. pylori* treatment in children is usually empiric and comprised of amoxicillin, clarithromycin, or metronidazole [15]. Typically, these antimicrobial agents are prescribed for other respiratory, genital, urinary, or even parasite pediatric infections [29]. Thus, ideal treatment should be tailored according to AST especially now the increasing antimicrobial resistance has become critical for the management of *H. pylori* infection.

The first step in our investigation was to summarize each patient’s characteristics and perform the UGE with biopsy collection for histopathological analysis and bacterial cultures. The second phase involved the assessment of the bacterial cultures with AST, and the third and final step comprised the therapeutic efficacy evaluation. 

The mean duration between the symptoms’ onset and the diagnosis for patients having a family history of upper GI disorders was shorter than for those without a family history but not statistically significant. However, our results may have been influenced by the limited number of patients in our study. Since family history represents an independent predictor of several GI diseases, the most severe being gastric cancer [30,31,32], it is fairly understood that these patients would seek medical attention sooner after the symptoms develop. Moreover, the endoscopic findings were milder in patients that received previous treatment when compared to the others, consistent with our previous results [33]. Six of seven patients with normal endoscopic mucosa received prior treatment. These results suggest that children might become “tolerant” to the bacterium or that the growing child is more resistant to *H. pylori*-induced lesions. The evidence that *H. pylori* infection in children coexists with normal gastric mucosa was also reported in other studies [11,33]. This is the reason why we strongly recommend taking biopsies at least for the histological exams in children and adolescents, even if a normal appearance of the mucosa is observed during endoscopy. 

Correct diagnosis, AST, and proper treatment of *H. pylori* gastric infection are pivotal for a positive outcome. The initial diagnosis should be made using invasive gastric biopsy techniques, including positive bacterial cultures, or histopathological findings of *H. pylori* with at least one other positive test (e.g., RUT, or molecular-based assays when available, including polymerase chain reaction or fluorescent in situ hybridization), according to the most recent guidelines for the management of *H. pylori* in children and adolescents. Non-invasive techniques such as ^13^C-UBT or stool antigens should not be used for the initial *H. pylori* diagnosis. However, when positive histology is the only available invasive test, a positive non-invasive result supports the diagnosis [15].

Fifty-nine out of sixty-eight patients enrolled in this study were diagnosed with *H. pylori* infection, indicating an 86.76% positivity rate. However, the positivity rate may be overestimated by the fact that all the patients initially presented a positive fecal antigen test. The histopathologic examination determined the diagnosis in 94.91% of the cases identifying the presence of *H. pylori* in 56 patients. The hematoxylin–eosin and Giemsa stains of the gastric antrum and body identified a higher number of patients with *H. pylori* infection compared to the bacterial cultures. The result was recorded as positive if the bacterium was isolated in either the antral mucosa and/or gastric body. The sensitivity and specificity of histopathology for the diagnosis depend on clinical settings, the density of colonization, and the histopathologist’s experience [34]. Histopathology delivers excellent diagnostic accuracy, allowing the detection of the bacteria, but also provides information about the gastritis grading. In children, the current recommended guideline to asses *H. pylori* histological changes in gastritis is the Updated Sydney Classification [16]. The usual recommendation derived from the Sydney system is to obtain two biopsy specimens from the antrum and two specimens from the corpus. Bacteria are usually present at both sites even if the lesions occur essentially in the antrum. Thus, other studies showed that only two antral biopsies were sufficient to detect *H. pylori* [35]. In our study, we determined a statistically significant correlation between the activity of gastritis and the *H. pylori* density in the histopathological exam. This significant difference allowed us to conclude that histologic *H. pylori* density was linked with the degree of gastritis activity. In comparison with adults, gastric biopsies obtained from children infected with *H. pylori* show a lower degree of inflammation with a predominant lymphocytic component [36]. In addition, a higher number of immunosuppressive regulatory T cells and a more prominent IL-10-mediated anti-inflammatory response, and lower IL-17 have been detected in pediatric patients [15,37]. In other studies, the strain of the organism may be a more important factor than the density of infection in determining the gastric inflammatory response to *H. pylori* [35,38]. Thus, children usually exhibit high colonization levels with scarce acute inflammation [11,36,37,39,40]. 

Only 13 out of 59 patients (22.03%) presented positive *H. pylori* cultures. These bacteria can be cultured from gastric biopsies, although this process frequently presents difficulties. *H. pylori* lose viability when exposed to the environment; thus, biopsies should be cultured quickly. If this is not possible, a specific transport media has to be used. Even experienced laboratories can culture the pathogen from 50% to 93% of the infected biopsies [34,41]. 

In our study, the specificity of *H. pylori* cultures was 93.87%, but the sensitivity was markedly decreased with a value of 22.03%. As the sensitivity of the culture method is low, a negative *H. pylori* culture does not indicate the absence of the infection. In the literature, various studies report inconsistent results. For example, Kaya et al. reported an *H. pylori* culture sensitivity of 22.5% and a specificity of 97.1% [42]. In another study conducted on children and adolescents, the sensitivity for *H. pylori* culture was 79.3% and the specificity was 100% [43]. In the adult population, the sensitivity of the culture method is higher, ranging from 62.7% to 96.3% in the performed studies [42,44,45]. Despite their long use, cultures are challenging due to the fastidious nature of the bacterium, with growth requirements regarding the media [46]. Therefore, although the culture method is accepted as the diagnostic “gold standard”, it is difficult to use solely as a routine diagnostic tool. The number of biopsies necessary to diagnose *H. pylori* infection is also a subject of controversy. Although a single antrum biopsy specimen (2 cm from the pylorus) has good sensitivity, it is not sufficient to provide a reliable diagnosis. The probability of finding *H. pylori* increases with the number of biopsy specimens tested since the bacterium may have a patchy distribution [47]. Although *H. pylori* are often present everywhere, there are some rare cases when the infection solely affects the corpus. For example, the corpus may be the only site that remains positive after the consumption of antisecretory drugs [19]. In our study, we only obtained two biopsies from the antrum for *H. pylori* cultures, and this could be adjusted by getting another one or two samples from the gastric body to improve the culture success rate. However, taking more biopsy samples can be difficult to apply to children.

To establish optimum therapy, meaning to achieve maximum efficacy and minimum side effects, it is essential to determine which antibiotic resistance has actually developed for every patient. Ideally, AST should be performed for all diagnosed patients. However, in standard clinical practice, AST is not always possible as it is challenging to use the culture method alone as a routine diagnostic approach. 

In our study, antibiotic resistance was encountered only in two of the patients with *H. pylori*-positive cultures, accounting for 15.38% of the total number of cultures. More specifically, they presented clarithromycin resistance. Both patients previously underwent treatment for *H. pylori* infection. The international guidelines do not recommend clarithromycin when resistance to this antibiotic is >15% [15], as in our case. 

A recent multicenter study across Europe evaluated *H. pylori* antibiotic resistance for a long period of time (i.e., 7 years, between 2013 and 2020) [48]. This study included 41,526 adult patients with bacterial cultures performed for 3974 (9.5%) individuals. This evidence suggests a low rate of using this method as it is not part of the routine clinical practice. The key finding was that only 9.5% of cases in Europe involved testing for antibiotic susceptibility, resulting in a low number of tests being conducted [48]. Our study reveals a low use of AST also in children (i.e., 22.03%). In the previously mentioned study, *H. pylori* resistance was generally high for clarithromycin, especially in countries in Southern Europe. The average resistance rate to clarithromycin was 25%, with a maximum peak in 2016 reaching 34% [48]. In our study, only clarithromycin resistance was encountered (i.e., 15.38%), presenting lower values but under the reserve of a limited number of positive cultures. Other studies also observed a significant increase in the *H. pylori* resistance rate toward clarithromycin, levofloxacin, and metronidazole which correlate with the number of previous treatment failures [49]. In another pediatric investigation, including 154 children, the highest amoxicillin resistance was reported, at 12%. The resistance rate for clarithromycin was 35%, and for tetracycline and levofloxacin was 8% and 2%, respectively [50]. 

Understanding *H. pylori* susceptibility to antibiotics and adherence to therapy is essential for successfully eradicating pediatric *H. pylori* infection [51]. At least a 90% eradication rate is the ideal target for first-line therapy, especially for preventing the emergence of antibiotic-resistant strains [14,52]. However, if culture and AST for *H. pylori* are unavailable, the treatment may rely on clinical experience and regional antimicrobial susceptibility profiles. In our study, the eradication rate for sequential therapy was successful, exceeding 90% (94.73%). This high efficacy of the sequential regimen is similar to a couple of other studies, reporting eradication rates ranging from 81.9% to 98% [53,54,55]. However, these eradication rates are lower when antibiotic resistance is present. For instance, sequential therapy efficacy was only 56% when clarithromycin resistance was present in a prospective multicenter European study of 165 children [56]. Thus, data collected from our study suggest a high susceptibility of *H. pylori* to antibiotics. This multi-drug treatment is particularly challenging for clinicians because pediatric patients must take pills several times a day. Thus, to improve compliance and prevent poor compliance or treatment discontinuation, which results in treatment failure, physicians should also educate patients’ families.

## 5. Study Limitations

We acknowledge that our study has several limitations. First of all, this is a single-center study, with a limited number of patients during a specific timeframe. This small number of patients restricted us to obtain possible significant results regarding antibiotic resistance. Moreover, the current pediatric guidelines recommend a diagnosis based on bacterial cultures or histology with at least one other positive test. However, when the study started the guidelines were not published yet. Thus, we did not perform in every case the extra needed test for the histopathological diagnosis. Furthermore, the guidelines propose obtaining at least six biopsy samples for the histopathological examination. Due to the fact that our patients were children and we also needed to collect samples for the bacterial cultures, we were unable to gather so many samples for our study. Therefore, in some of these circumstances, a misdiagnosis may be linked to our detecting methods. Additionally, sequential therapy effectiveness could be overestimated because this therapy was only prescribed to patients with no antibiotic resistances or negative cultures.

## 6. Conclusions

The encountered impediments in the bacterial growth in cultures restricted us from further accurately investigating the *H. pylori* antibiotic resistance. The histopathologic examination brought more information and was more easily performed. Thus, our recommendation is to continue using for the diagnosis biopsy-based investigations such as histopathology, but also determine the bacterial cultures with AST if possible or depending on local availability. Although the bacterial cultures had a low positivity rate, the sequential therapy had a successful eradication rate, suggesting the susceptibility of *H. pylori* infection in the children from our center with previous AST analysis.

To summarize, our data recommend that further clinical studies are necessary to better assess the *H. pylori* antibiotic resistance to provide tailored treatment and identify children that need closer monitoring.

## Figures and Tables

**Table 1:** Demographic and clinical characteristics of the patients.

Mean age ± SD, years	11 years 7 months ± 4 years 5 months
Male/female	24 (40.67%)/35 (59.32%)
Family history for *H. pylori* infection	10/59 (16.94%)
Symptoms > 6 months	26/59 (44.06%)
Previous therapy	22/59 (37.28%)

**Table 2:** Correlation between endoscopic aspect and previous *H. pylori* eradication status.

Parameter	Normal Endoscopic Aspect	Pathological Endoscopic Aspect	*p* Value
Patients without previous therapies (37 patients)	1/37 (2.70%)	36/37 (97.29%)	0.002
Patients with previous therapies (22 patients)	7/22 (31.81%)	15/22 (68.18%)	

**Table 3:** Correlation between *H. pylori* density in the histological examination, positive bacterial cultures, and the activity of associated gastritis.

Parameter		Low *H. pylori* Density	Moderate *H. pylori* Density	Marked *H. pylori* Density	*p* Value
Positive *H. pylori* cultures (%)		5/28 (17.85%)	4/21 (19.04%)	1/7 (14.28%)	0.01
Activity of *H. pylori* gastritis	Without activity (%)	7/28 (25%)	1/21 (4.76%)	1/7 (14.28%)	0.03
	Mild activity (%)	11/28 (39.2%)	6/21 (28.57%)	4/7 (57.14%)	
	Moderate activity (%)	10/28 (35.7%)	13/21 (61.9%)	2/7 (28.57%)	
	Severe activity (%)	0	1/21 (4.76%)	0	
